# New Drugs and Preparations

**Published:** 1894-11-10

**Authors:** 


### NEW DRUGS AND PREPARATIONS.

### ACTION OF SALICYLIC ACIDS.

Professor Stokvis, of Amsterdam, read a paper at the International Medical Congress on the action of salicylic acids prepared from three different sources, viz., from anthranilic acid (called here the A. acid), from oil of winter-green (the W. acid), and from phenol (the P. acid). The chemical characters of all three acids are identical, but they differ physiologically, the W. acid being more quickly eliminated, and being a more active diuretic than the others. The latter, however, caused tremors, or sometimes even convulsions, which the W. acid never did. In other respects also the acid from oil of winter-green was found to be less poisonous than the P. or A. acids. Further chemical examination showed the A. and P. acids to be quite pure, while the W. acid contained salicylate of methyl. Stokvis holds that the pure acids are more poisonous than the impure, and that the impurity of the salicylic acid made from oil of winter-green confers on it a comparative absence of toxic effects. This result, however, is contrary to that of Professor Charteris, of Glasgow, who found that the natural salicylic acid was less poisonous than that made from phenol.

### LACTOPHENIN.

This body is phenacetin in which the acetyl radical is replaced by the radical of lactic acid. It forms a white powder with little taste, and soluble in 330 parts of water. A medium dose is 10 grains thrice daily, a large dose 15 grains. Landowski (Bull. Med. VIII., 125) has found it to be analgesic, and in large amount hypnotic in action. It is well tolerated by the stomach and otherwise, but like all similar substances has a tendency to cause perspiration. It has been used in rheumatism, influenza, scarlet fever, and other conditions, with results very similar to those obtained from phenacetin or phenazone. Von Jaksch (Centbl. f. innere Med., 1894, No. II.) has used it with good results in the treatment of typhoid fever. Temperature was gradually and satisfactorily reduced, and collapse, cyanosis, excessive perspiration, or rigors are not caused by it. The author was particularly pleased with its sedative effect on his patients: the delirium subsides, consciousness is restored, there is a feeling of comfort and greater well-being, and the appetite is improved. In all, 18 cases were treated, and Von Jaksch was so much impressed by its value that he thinks it may advantageously replace the cold bath treatment in many cases. He is careful to point out, however, that it is in no sense curative, but is simply a good means of modifying the patient's condition and symptoms.

